# A new species of *Chucallis* Tao (Hemiptera, Aphididae, Calaphidinae) from China

**DOI:** 10.3897/zookeys.146.2042

**Published:** 2011-11-09

**Authors:** Li-Yun Jiang, Jing Chen, Ge-Xia Qiao

**Affiliations:** 1 Key Laboratory of Zoological Systematics and Evolution, Institute of Zoology, Chinese Academy of Sciences, No. 1 Beichen West Road, Chaoyang District, Beijing 100101, P.R.China; 2 Graduate University of Chinese Academy of Sciences, No. 19, Yuquan Road, Shijingshan District, Beijing 100049, P.R.China

**Keywords:** *Chucallis*, Aphididae, Calaphidinae, new species, China

## Abstract

A new species in the aphid genus *Chucallis* Tao, *Chucallis latusigladius* Qiao, Jiang & Chen, sp. n. feeding on a species of bamboo, *Indocalamus tessellatus* (Munro) Keng f., is described. It differs from the only other known species in the genus by having remarkably large marginal processes on abdominal tergite IV. A key to species, morphological descriptions, distributional data and host plant information are provided. The type specimens studied are deposited in the National Zoological Museum of China, Institute of Zoology, Chinese Academy of Sciences, Beijing, China.

## Introduction

The aphid genus *Chucallis* was erected by Tao (1964), based on *Myzocallis bambusicola* Takahashi, 1921 as the type species. The genus can be easily separated from related genera by having long, finger-like dorsal processes on the abdomen, especially marginal ones. Until now, only one species is known (Remaudière and Remaudière 1997). After identifying the specimens and checking the specimens of the type species, we found a new species, *Chucallis latusigladius* Qiao, Jiang & Chen, sp. n., which is described in this paper. It is different from the only other known species in the genus in having remarkably large marginal processes on abdominal tergite IV. It feeds on one species of bamboo, *Indocalamus tessellatus* (Munro) Keng f., and occurs in Zhejiang and Fujian, China.

## Materials and methods

Aphid terminology generally follows Quednau (2003) and Qiao et al. (2005). The unit of measurements is millimeters (mm). Metrical data were listed in Table 1.

**Table 1.** Metrical data of *Chucallis latusigladius* Qiao, Jiang & Chen, sp. n.

Parts*		**Alate viviparae (n=13)**		
		**Mean**	**Range**	**Standard Deviation**
	Body length	2.112	1.670–2.266	0.122
	Body width	0.855	0.691–0.96	0.048
	Whole Antennae	1.805	1.670–1.901	0.056
	Ant.I	0.076	0.067–0.086	0.003
	Ant.II	0.058	0.058	0
	Ant.III	0.575	0.461–0.614	0.035
	Ant.IV	0.333	0.288–0.365	0.021
	Ant.V	0.335	0.317–0.374	0.014
	Ant.VIb	0.178	0.173–0.182	0.005
	PT	0.224	0.211–0.259	0.010
	URS	0.08	0.077–0.096	0.004
	Hind femur	0.588	0.48–0.643	0.029
	Hind tibia	1.113	0.974–1.210	0.045
	2HT	0.095	0.086–0.106	0.005
	SIPH	0.160	0.134–0.202	0.017
Length (mm)	SIPH BW	0.193	0.134–0.230	0.021
	SIPH DW	0.073	0.067–0.077	0.004
	Cauda	0.135	0.115–0.144	0.005
	BW Cauda	0.162	0.154–0.192	0.010
	Ant.IIIBW	0.019	0.019	0
	MW Hind tibia	0.032	0.024–0.038	0.004
	MT on Tergum IV	0.68	0.547–0.749	0.041
	BW of MT on Tergum IV	0.166	0.144–0.192	0.016
	SW of MT on Tergum IV	0.126	0.096–0.154	0.017
	DW of MT on Tergum IV	0.050	0.038–0.077	0.013
	Cephalic setae	0.042	0.038–0.058	0.007
	Setae on Tergum I	0.041	0.029–0.058	0.007
	Setae on Tergum VIII	0.039	0.029–0.058	0.007
	Setae on ANT.III	0.014	0.014	0
	Setae on SIPH	0.092	0.067–0.115	0.010
	Setae on MT of Tergum IV	0.030	0.019–0.029	0.005
	Setae on Hind tibia	0.046	0.038–0.058	0.005
	Whole Antennae / Body	0.83	0.79–0.88	0.025
	Hind femur / Ant.III	1.03	0.98–1.09	0.029
	Hind tibia / Body	0.54	0.50–0.59	0.020
	PT / Ant.VIb	1.26	1.16–1.33	0.051
	URS / BW URS	1.04	0.89–1.14	0.069
	URS / 2HT	0.86	0.72–1.11	0.075
	Cauda / BW Cauda	0.83	0.70–0.94	0.070
	Cephalic setae / Ant.IIIBW	2.13	1.50–3.00	0.324
	Setae on Tergum I / Ant.IIIBW	2.13	1.50–3.00	0.378
Ratio (times)	Setae on Tergum VIII / Ant.IIIBW	2.08	1.50–3.00	0.352
	Setae on ANT.III / ANT.IIIBW	0.75	0.75	0
	Setae on hind tibia / MW Hind tibia	1.46	1.25–1.67	0.146
	SIPH / Cauda	1.20	1.00–1.43	0.139
	SIPH / SIPH BW	0.83	0.68–1.11	0.091
	SIPH / SIPH DW	2.09	1.67–2.86	0.274
	MT on Tergum IV / Ant.V	2.06	1.69–2.19	0.130
	Setae on SIPH / SIPH DW	1.26	0.88–1.50	0.112
	Setae on MT of Tergum IV / Ant.IIIBW	1.54	1.00–2.00	0.261

* Abbreviations. Ant. I, II, III, IV, V, VIb, antennal segments I, II, III, IV, V and the base of antennal segment VI, respectively; PT, processus terminalis; Ant.IIIBW, the basal width of antennal segment III; URS, ultimate rostral segment; BW URS, basal width of ultimate rostral segment; 2HT, second hind tarsal segment; MW hind tibia, mid-width of hind tibia; SIPH, siphunculi; SIPH BW, basal width of siphunculi; SIPH DW, distal width of siphunculi; MT on Tergum IV, marginal tubercle on abdominal tergite IV; BW of MT on Tergum IV, basal width of marginal tubercle on abdominal tergite IV; SW of MT on Tergum IV, width of marginal tubercle on abdominal tergite IV where seta distributing; DW of MT on Tergum IV, distal width of marginal tubercle on abdominal tergite IV; BW Cauda, basal width of cauda.

## Taxonomy

### Chucallis

Tao

http://species-id.net/wiki/Chucallis

Chucallis Tao, 1964: 221. Type species: *Myzocallis bambusicola* Takahashi, 1921; by monotypy.
Chucallis Tao: Tao 1990: 139; Zhang 1999: 227–228; Qiao et al. 2005: 184.

#### Generic diagnosis.

In alate viviparae, frontal tubercle not developed. Head without epicranial suture, clypeus without any processes. Antennae 6-segmented, processus terminalis slightly longer than base of the segment. Head and thorax without any dorsal processes. Abdominal tergites with dorsal spinal processes and developed marginal processes. Dorsal setae of body long and pointed, thick or fine. Rostrum short and stout. Wing veins without black borders. Fore coxae distinctly expanded, mid- and hind coxae normal. First tarsal chaetotaxy: 5, 5, 5. Siphunculi truncated, slightly longer than their basal diameters. Cauda knob-shaped. Anal plate bilobed. Gonapophyses fused.

In embryos, dorsal setae of body thick and long, pointed or stout. Dorsum of head with 2 pairs of anterior and 2 pairs of posterior setae. Thoracic tergites each with 1 pair of spinal and 1 pair of marginal setae, respectively. Abdominal tergites I–VII each with 1 pair of spinal and 1 pair of marginal setae, respectively, and spinal setae on tergites III, V and VII slightly displaced pleurally; tergite VIII with 2 dorsal setae. Siphunculi visible.

#### Distribution.

Only found in China (Zhejiang, Fujian, Sichuan, Gansu, Taiwan and Hong Kong).

**Host plants.** Plants of Gramineae/Poaceae,feeding on bamboos,such as *Bambusa stenostachya* Hack., *Dendrocalamus latiflorus* Munro, *Phyllostachys heteroclada* Oliv., *Thamnocalamus spathaceus* (Franch.) Soderstr. and *Indocalamus tessellatus*.

#### Biology.

The species colonize the undersides of the leaves of their host plants.

#### Comments.

The genus is similar to *Subtakecallis* Raychaudhuri & Pal and *Takecallis* Matsumura (Quednau 2003), but the alatae differ from those genera in having the clypeus without a nose-like processus (*Subtakecallis* and *Takecallis*: the clypeus with a nose-like processus), and some abdominal tergites with long, finger-like, seta-bearing spinal and marginal processes, the marginal ones on tergite IV being especially large (*Subtakecallis* and *Takecallis*: some abdominal tergites with small processes, not longer than their basal width).

#### Key to species of *Chucallis*

(Alate viviparous females)

**Table:** 

1	Body dark purple to black in life; marginal processes on abdominal tergite IV about 0.60 times as long as antennal segment V; abdominal tergite VIII with 4 setae; siphunculi shorter, about 0.7 times as long as cauda	*Chucallis bambusicola* (Takahashi)
–	Head and thorax pale brown, abdomen dark green in life; marginal processes on abdominal tergite IV 1.69–2.19 times as long as antennal segment V; abdominal tergite VIII with 5 or 6 setae, occasionally 7 or 8 ones; siphunculi longer, 1.00–1.43 times as long as cauda	*Chucallis latusigladius* Qiao, Jiang & Chen, sp. n.

### Chucallis bambusicola

(Takahashi)

http://species-id.net/wiki/Chucallis_bambusicola

Myzocallis bambusicola Takahashi 1921: 70
Agrioaphis bambusicola Takahashi 1931: 85; Tseng and Tao 1938: 209.
Chucallis bambusicola (Takahashi): Tao 1964: 62; Tao 1990: 139; Zhang 1999: 228; Qiao et al. 2005: 184–186.

#### Specimens examined.

2 alate viviparous females, 4 May 1975, Zhejiang (Hangzhou City), No. 5567, on *Phyllostachys heteroclada*, coll. T.S. Zhong; 1 alate viviparous female, 7 August 1987, Gansu (Chongxin County), No. 8858, on a kind of bamboo, coll. T.S. Zhong; 2 alate viviparpus females and 2 nymphs, 25 July 1986, Gansu (Longxi County), No. 8507, on a kind of bamboo, coll. G.X. Zhang, T.S. Zhong & J.H. Li; 4 alate viviparpus females and 6 nymphs, 22 July 1985, Gansu (Tianshui City, Maijishan Mountain), No. 8092, on a kind of bamboo, coll. G.X. Zhang & T.S. Zhong; 8 alate viviparpus females and 3 nymphs, 1 August 1987, Gansu (Gangu County), No. 8819, on *Thamnocalamus spathaceus*a, coll. T.S. Zhong.

#### Distribute.

CHINA: Zhejiang (Hangzhou), Gansu (Chongxin, Longxi, Tianshui, Gangu), Sichuan (Chengdu), Taiwan and Hong Kong (Tao 1990).

#### Biology.

On shoots and undersides of leaves of bamboos (*Bambusa stenostachya*, *Dendrocalamus latiflorus*, *Phyllostachys heteroclada*, *Thamnocalamus spathaceus*).The species is very active, jumping when disturbed. Anholocyclic in Taiwan (Takahashi 1921), and sexual morphs are unrecorded (Blackman and Eastop 1994).

### Chucallis latusigladius

Qiao, Jiang & Chen sp. n.

urn:lsid:zoobank.org:act:2A359DA9-53F8-4D15-8348-41588AC1AF9D

http://species-id.net/wiki/Chucallis_latusigladius

**Figures 1–18.**
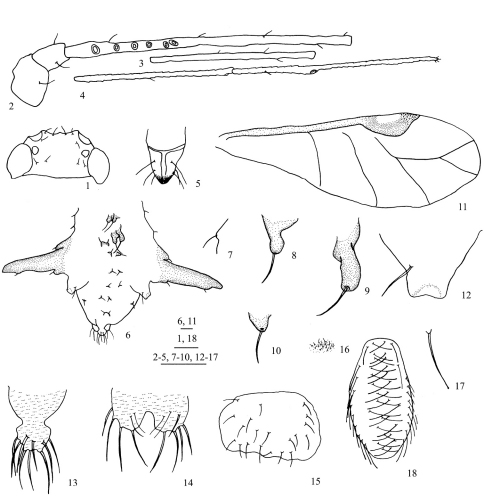
*Chucallis latusigladius* Qiao, Jiang & Chen, sp. n. Alate viviparous female: **1** dorsal view of head **2** antennal segments I–III **3** antennal segment IV **4** antennal segments V–VI **5** ultimate rostral segment **6** dorsal view of abdomen, showing processes **7** marginal tubercle on abdominal tergite I **8** spinal tubercle on abdominal tergite I **9** spinal tubercle on abdominal tergite II **10** spinal tubercle on abdominal tergite III **11** fore wing **12** siphunculus **13** cauda, **14** anal plate, **15** genital plate **16** gonapophyses. Embryo: **17** dorsal seta of body **18** setal pattern. Scale bars = 0.10 mm.

Figures 1 [Fig F2] [Fig F3] [Fig F4] –42

#### Locus typicus.

China (Zhejiang, 28.39533°N, 118.84490°E, altitude 450m).

#### Etymology.

The species is named for the very large, broadsword-shaped marginal processes on abdominal tergite IV. The specific name combines “*latus* (Latin, =broad, wide)” and “*gladius* (Latin, =sword)”.

#### Descriptions.

**Figures 19–30. F2:**
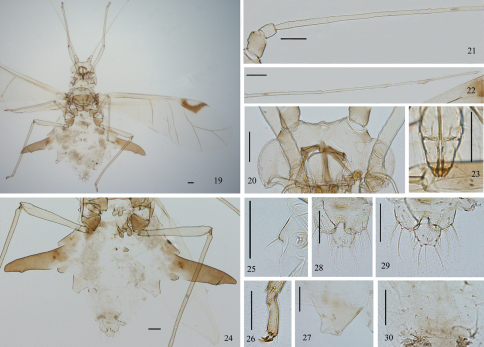
*Chucallis latusigladius* Qiao, Jiang & Chen, sp. n. Alate viviparous female: **19** dorsal view of body **20** dorsal view of head **21** antennal segments I–III **22** antennal segments IV–VI **23** ultimate rostral segment **24** dorsal view of abdomen **25** marginal tubercle and seta on abdominal tergite I **26** hind tarsal segments **27** siphunculus **28** cauda **29** anal plate **30** genital plate. Scale bars = 0.10 mm.

*Alate viviparous female*: Body oval (Fig. 19), head and thorax pale brown, abdomen dark green in life (Figs. 36–38).

**Mounted specimens.** Head and thorax pale brown, dorsal spinal processes, marginal processes and seta-bearing processes brown; antennal segment I pale brown, antennal segments II-VI unpigmented; apex of rostrum brown; femora, tibiae and tarsi pale brown; siphunculi, cauda, anal plate and genital plate pale brown; wing veins pale and unbordered, basal and inner margins of pterostigma with dark fuscous forming a conspicuous crescent-shaped mark (Fig. 19); the other parts of specimens pale. Posterior margin of pronotum with short wrinkles; distal 1/3 of antennal segment III and segments IV–VI with sparse imbrications, middle of inner margin of segment I slightly swollen (Figs. 2, 21); distal 1/4 of tibiae with spinules, tarsi with spinulose short imbrications; dorsal spinal and marginal processes with sparse spinules or spinulose short stripes; cauda, anal plate and genital plate with sparse spinulose short stripes. Dorsal setae of body thick and pointed, long or short. Head with 1 pair of cephalic setae, 2 pairs of antennal tubercular setae and 2 pairs of posterior dorsal setae between eyes (Fig. 1); pronotum with 2 pairs of spinal and 1 pair of posterior marginal setae; abdominal tergite I with 1 pair of spinal and 1 pair of marginal setae, each on dorsal processes; tergite VIII with 5 or 6 setae, occasionally 7 or 8, 1 pair of them on dorsal spinal processes. Length of cephalic setae, marginal setae on abdominal tergite I and dorsal setae on tergite VIII 0.038–0.058, 0.029–0.058 and 0.029–0.058, respectively, all 1.50–3.00 times as long as basal width of antennal segment III.

**Head.** Median front and antennal tubercles un-developed, frontal profile shallow “W”-shaped (Figs. 1, 20). Dorsum of head without any processes. Antennae fine and long (Figs. 2–4, 21, 22), 6-segmented, 0.79–0.88 times as long as body; length in proportion of segments I–VI: 12 : 9 : 100 : 60 : 60 : 36+47, respectively; processus terminalis 1.16–1.33 times as long as base of the segment. Secondary rhinaria round, with sparse and short ciliated, 4–7 ones distributing on basal 1/3–2/5 of antennal segment III. Antennal setae short and pointed, segments I–VI each with 2 or 3, 2 (occasionally 1), 5–7 (occasionally 3 or 4), 1–3, 1 (occasionally 2), 1+0 setae, respectively; apex of processus terminalis with 4 or 5 short pointed setae; setae of segment V distributing on basal 1/3 of the segment; length of setae on antennal segment III 0.75 times as long as basal width of the segment. Rostrum thick and short, apex reaching anterior margin of mesosternum; ultimate rostral segment stout, wedge-shaped (Figs. 5, 23), 0.89–1.14 times as long as its basal width, 0.72–1.11 times as long as second hind tarsal segment, with 3 pairs of primary and 3 pairs of accessory setae.

**Thorax.** Dorsum of thorax without any processes. Legs slender. Fore coxae distinctly expanded, mid- and hind coxae normal. Hind femur 0.98–1.09 times as long as antennal segment III, hind tibia 0.50–0.59 times as long as body. Setae on legs short, stiff and pointed, apex of tibiae with 3 peg-shaped setae (Fig. 26), distinct differ from other setae. Length of setae on hind tibia 1.25–1.67 times as long as mid-width of the segment. First tarsal chaetotaxy: 5, 5, 5. Wing veins pale without bordered; forewing with radial sector absent or with basal half indistinct; basal and inner margins of pterostigma thickly marked with brown fuscous (Figs. 11, 19); hind wings with two oblique veins.

**Abdomen.** Abdominal tergites with developed spinal and marginal processes (Figs. 6, 24). Tergite I: 1 pair of conical spinal processes (Fig. 8), 0.03–0.08 long, 0.57–1.38 times as long as its basal width, 0.75–2.75 times as long as its distal width; apex of each tubercle with 1 thick, long and pointed seta, 0.06–0.14 long, 1.17–2.14 times as long as length of spinal processes; marginal processes not well developed (Figs. 7, 25), 0.019–0.029 long, 0.50 times as long as their basal widths. Tergite II: 1 pair of long conical spinal processes (Fig. 9), 0.096–0.173 long, 0.73–1.64 times as long as their basal widths, 2.50–4.50 times as long as their distal widths; a spinal seta on apex of each spinal tubercle, 0.067–0.086 long, 0.60–0.67 times as long as length of spinal processes; marginal processes conical, 0.029–0.048 long, 0.25–0.50 times as long as their basal widths; marginal setae as long as marginal processes. Tergite III: 1 pair of spinal processes (Fig. 10), which are close to each other at base, 0.019–0.048 long, 0.75–0.80 times as long as their basal widths; a spinal seta on apex of each spinal processus, 0.038–0.086 long, 1.60–3.00 times as long as length of spinal processes; 1 pair of pleural setae not on processes; marginal processes 0.106–0.182 long, 0.59–1.19 times as long as their basal widths; marginal setae 0.029–0.048 long, 0.16–0.30 times as long as marginal processes. Tergite IV: 1 pair of spinal processes, 0.048–0.086 long, 0.71–1.50 times as long as its basal width; spinal setae short and pointed, 0.028–0.048 long, about 0.50 times as long as spinal processes; marginal processes distinctly developed, broadsword-shaped, almost vertical with body in life (Figs. 36–38); 1.69–2.19 times as long as antennal segment V, 3.75–4.73 times as long as their basal widths; each with 1 seta on distal 1/3 of inner margin, 0.019–0.029 long, 1.00–2.00 times as long as basal width of the antennal segment III. Tergite V: 1 pair of spinal processes, 0.010–0.048 long, 0.25–1.67 times as long as their basal widths; spinal setae 0.048 long, 0.20–1.00 times as long as spinal processes; marginal processes 0.048–0.067 long, 0.45–0.78 times as long as their basal widths, marginal setae 0.058–0.077 long, 0.86–1.60 times as long as marginal processes, sometimes with 1 pair of short and pointed pleural setae on small processes. Tergite VI: 1 pair of spinal processes, 0.019–0.029 long, 0.43–0.67 times as long as their basal widths; each with 1 seta at apex, 0.029–0.038 long, 1.00–2.00 times as long as spinal processes. Tergite VII: 1 pair of spinal processes, 0.01–0.019 long, 0.33–0.50 times as long as their basal widths; 1 pair of spinal setae on spinal processes, 0.048–0.058 long, 2.50–3.00 times as long as spinal processes; 1 pair of marginal processes, 0.029–0.048 long, 0.50–0.72 times as long as their basal widths, marginal setae on marginal processes 0.048–0.077 long, 2.50–4.00 times as long as basal width of antennal segment III; 1 pair of pleural setae not on processes. Tergite VIII: 1 pair of spinal processes, 0.010–0.038 long, 0.33–1.00 times as long as their basal widths; pleural and marginal setae not on processes. Siphunculi truncated (Figs. 12, 27), smooth, without flange, 0.68–1.11 times as long as their basal widths, 1.67–2.86 times as long as their distal widths, 1.00–1.43 times as long as cauda; each with 1 long seta at base of siphunculi, length of seta 0.88–1.50 times as long as distal width of siphunculi**.** Cauda knob-shaped (Figs. 13, 28), 0.70–0.94 times as long as its basal width; with 9–12 long or short setae. Anal plate bilobed (Figs. 14, 29), with 18–23 long or short setae. Genital plate transversely oval (Figs. 15, 30), with 20–32 fine setae. Gonapophyses fused, with 11–15 short pointed setae (Fig. 16).

*Embryo(in alate viviparous female)*. Dorsal setae of body thick, long and capitate at apex, with distinct basal processes (Fig. 17). Setal pattern (Fig. 18): dorsum of head with 2 pairs of anterior and 2 pairs of posterior setae; pro-, meso- and metanotum each with 1 pair of spinal and 1 pair of marginal setae, respectively; abdominal tergites I–VII each with 1 pair of spinal and 1 pair of marginal setae, respectively; spinal setae on tergites III, V and VII slightly displaced pleurally; tergite VIII with 2 spinal setae. Siphunculi visible.

**Figures 31–34. F3:**
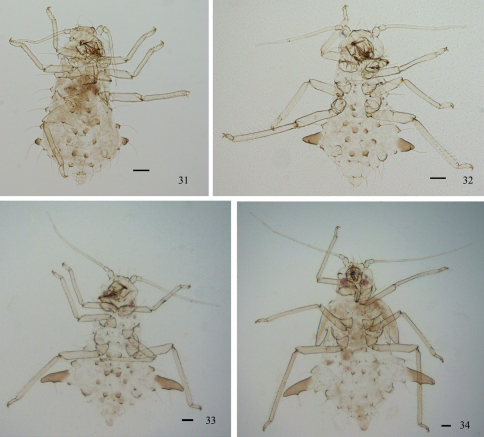
*Chucallis latusigladius* Qiao, Jiang & Chen, sp. n. Dorsal view of body: **31** 1^st^ instar nymph **32** 2^nd^ instar nymph **33** 3^rd^ instar nymph **34** 4^th^ instar nymph. Scale bars = 0.10 mm.

*First instar nymph.* Body oval, head and thorax yellow green, and abdomen dark green in life (Fig. 39). Mounted specimens pale, with brown dorsal processes (Fig. 31). Body 0.80–1.06 long and 0.50–0.62 wide. Antennae 4-segmented, segment III 0.208–0.213 long, basal diameter of the segment 0.0144–0.0192. Abdominal tergites I–VII each with 1 pair of spinal and 1 pair of marginal processes, respectively; spinal processes on tergites III, V and VII slightly displaced pleurally; tergite VIII with 2 spinal processes. Each processus with one seta, thick, long and capitate at apex, same as setae of embryos. Spinal processes on tergite II 0.017–0.022 long, with setae 0.095–0.110 long; marginal processes on tergite IV 0.05–0.06 long. Siphunculi truncated. Cauda triangle, with blunt round apex. Anal plate semicircle.

*Second instar nymph.* Body 1.02–1.27 long and 0.75–0.94 wide (Figs. 32, 40). Antennae 5-segmented, segment III 0.20–0.30 long, basal diameter of the segment 0.0144–0.0192. Spinal processes on tergite II 0.018–0.038 long, with setae 0.087–0.136 long; marginal processes on tergite IV 0.12–0.16 long. The other characteristics similar to first instar nymph.

*Third instar nymph.* Body 1.31–1.66 long and 1.11–1.43 wide (Figs. 33, 41). Antennae 6-segmented, segment III 0.21–0.25 long, basal diameter of the segment 0.0192–0.024. Spinal processes on tergite II 0.04–0.05 long, with setae 0.137–0.143 long; marginal processes on tergite IV 0.24–0.31 long. The other characteristics similar to first instar nymph.

*Fourth instar nymph.* Body 1.72–2.06 long and 1.57–1.86 wide (Figs. 34, 42). Antennae 6-segmented, segment III 0.37–0.40 long, basal diameter of the segment 0.0192–0.024. Spinal processes on tergite II 0.05–0.08 long, with setae 0.14–0.18 long; marginal processes on tergite IV 0.36–0.42 long. The other characteristics similar to first instar nymph.

#### Specimens examined.

Holotype: alate viviparous female, **CHINA**: Zhejiang (Suichang County, Jiulongshan Mountain, 28.39533°N, 118.84490°E, altitude 450m), 4 June 2011, No. 26816–1–1–1, on *Indocalamus tessellatus*, coll. J. Chen, Q.H. Liu & X.T. Li. Paratypes: 8 alate viviparous females, 2 first instar, 6 second instar, 4 third instar and 6 fourth instar nymphs, with the same collection data as holotype; 4 alate viviparous females, **CHINA**: Fujian (Jiangle County, Bailian District, Yujiaping Village, Longqishan Mountain, 26.52045°N, 117.30568°E, altitude 890m), 17 July 2011, on *Indocalamus tessellatus*, coll. J. Chen, Q.H. Liu & X.T. Li.

Specimen depositories. All the type specimens of the new species and the other specimens examined are deposited in the National Zoological Museum of China, Institute of Zoology, Chinese Academy of Sciences, Beijing, China.

#### Taxonomic notes.

The new species is similar to the type species *Chucallis bambusicola* (Takahashi), but differs in colour in life and morphology by the characters given in the key.

#### Host plant.

*Indocalamus tessellatus*.

#### Biology.

**Figures 35–38. F4:**
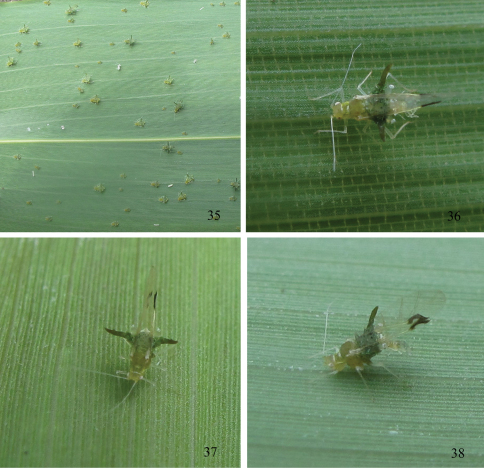
*Chucallis latusigladius* Qiao, Jiang & Chen, sp. n. **35** A population on the underside of leaf of host plant **36–38** Dorsal view of alate viviparous female in life.

It **c**olonizes the underside of the leaves of the host plant (Fig. 35).

**Figures 39–42.**
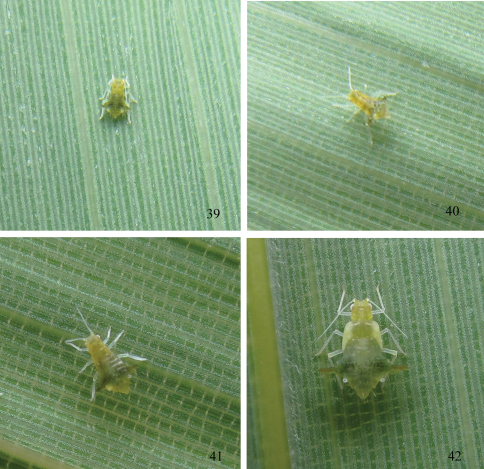
*Chucallis latusigladius* Qiao, Jiang & Chen, sp. n. Dorsal view of nymph in life: **39** 1^st^ instar nymph **40** 2^nd^ instar nymph **41** 3^rd^ instar nymph **42** 4^th^ instar nymph.

## Supplementary Material

XML Treatment for Chucallis

XML Treatment for Chucallis bambusicola

XML Treatment for Chucallis latusigladius
